# Salidroside Reduces Cell Mobility via NF-****κ****B and MAPK Signaling in LPS-Induced BV2 Microglial Cells

**DOI:** 10.1155/2014/383821

**Published:** 2014-04-17

**Authors:** Haixia Hu, Zuanfang Li, Xiaoqin Zhu, Ruhui Lin, Lidian Chen

**Affiliations:** ^1^Academy of Integrative Medicine Biomedical Research Center, Fujian University of Traditional Chinese Medicine, Huatuo Road, Minhou Shangjie, Fuzhou, Fujian 350108, China; ^2^Fujian Key Laboratory of Integrative Medicine on Geriatrics, Fujian University of Traditional Chinese Medicine, Huatuo Road, Minhou Shangjie, Fuzhou, Fujian 350108, China; ^3^College of Rehabilitation Medicine, Fujian University of Traditional Chinese Medicine, 1 Huatuo Road, Minhou Shangjie, Fuzhou, Fujian 350108, China

## Abstract

The unregulated activation of microglia following stroke results in the production of toxic factors that propagate secondary neuronal injury. Salidroside has been shown to exhibit protective effects against neuronal death induced by different insults. However, the molecular mechanisms responsible for the anti-inflammatory activity of salidroside have not been elucidated clearly in microglia. In the present study, we investigated the molecular mechanism underlying inhibiting LPS-stimulated BV2 microglial cell mobility of salidroside. The protective effect of salidroside was investigated in microglial BV2 cell, subjected to stretch injury. Moreover, transwell migration assay demonstrated that salidroside significantly reduced cell motility. Our results also indicated that salidroside suppressed LPS-induced chemokines production in a dose-dependent manner, without causing cytotoxicity in BV2 microglial cells. Moreover, salidroside suppressed LPS-induced activation of nuclear factor kappa B (NF-**κ**B) by blocking degradation of I**κ**B**α** and phosphorylation of MAPK (p38, JNK, ERK1/2), which resulted in inhibition of chemokine expression. These results suggest that salidroside possesses a potent suppressive effect on cell migration of BV2 microglia and this compound may offer substantial therapeutic potential for treatment of ischemic strokes that are accompanied by microglial activation.

## 1. Introduction

Inflammation has recently been implicated as a critical mechanism responsible for the progressive nature of neurodegeneration. It is now acknowledged that inflammation plays a major pathophysiological role in ischemic stroke [1]. Early reports described the brain as an immune privileged organ, but microglia are the resident innate immune cells in the central nervous system and undergo dramatic morphologic alterations upon activation, changing from resting ramified microglia into activated amoeboid microglia [2], releasing a variety of soluble factors, which are proinflammatory cytokines and chemokines which play a critical role in the inflammatory response associated with postischemia. The delayed inflammation provides a relatively wide and promising therapeutic window for intervention.

Salidroside, a phenylpropanoid glycoside separated from a medicinal plant* Rhodiola rosea*, had been reported that it exerted an anti-inflammatory effect on LPS-induced responses accompanied by the expression of pro-inflammatory cytokine involving MAPK signaling in microglial cells [3]. However, the role of salidroside has not been well elucidated in microglial mobility. This study investigated whether salidroside was able to inhibit the microglial migratory ability and the underlying molecular mechanisms responsible for the antichemotaxic activity of microglial BV cell.

## 2. Materials and Methods

### 2.1. Reagents

LPS (*Escherichia coli*, 055:B5) and MTT were obtained from Sigma (St. Louise, MO, USA). Salidroside (purity >99%) was purchased from the National Institute for the Control of Pharmaceutical and Biological Products (Beijing, China). The stock and working concentrations of salidroside were made by dissolving the powder in culture media to a concentration of 10 mM and 75, 150, and 300 *μ*M. The anti-murine monoclonal antibodies against ERK, phospho- (p-) ERK, JNK, p-JNK, p38, and p-p38 were purchased from Santa Cruz Biotechnology (Santa Cruz Biotechnology, CA). I*κ*B*α*, p-I*κ*B*α*, p65, p-p65, *β*-actin, and horseradish peroxidase- (HRP-) conjugated secondary antibodies were purchased from Cell Signaling (Beverly, MA). DMEM, FBS, and PBS were obtained from GIBCOBRL (Gaithersburg, MD, USA). Cytokines (MCP-1, MIP-1*α*, and IL-8) and ELISA kit were purchased from R & D Systems (Minneapolis, MN, USA).

### 2.2. Cell Culture and Treatment

The BV2 cells were maintained in a humidified incubator with 5% CO_2_ at 37°C and were cultured in high-glucose DMEM supplemented with 10% FBS, 100 *μ*g/mL streptomycin, and 100 U/mL penicillin. The BV2 cells were treated with LPS (1 *μ*g/mL) in the absence or presence of 75, 150, and 300 *μ*M salidroside, followed by exposure to serum-free DMEM culture medium for 4 h at 37°C in a humidified atmosphere of 95% air and 5% CO_2_. BV2 cells were grown in 96-, 24-, and 6-well plates at the concentration of 8 × 10^4^, 1 × 10^5^, and 5 × 10^5^ cells/well, respectively, in the indicated treatment for BV2 culture. At the end of cell treatments, we carried out the following different tests.

### 2.3. MTT Viability Assay

MTT assay was used as the indicator for effect of different concentrations of salidroside viability. Briefly, BV2 cells were incubated with salidroside at concentrations of 75, 150, and 300 *μ*M and/or LPS (1 *μ*g/mL) for 24 h, followed by serum-free DMEM culture as demonstrated before. After various treatments, the medium was replaced with 0.5 mg/mL of MTT solution and were incubated for 4 h at 37°C and 5% CO_2_. Afterwards, 100 *μ*L of DMSO solution was added to each well to dissolve the formazan and the absorbance was measured by spectrophotometry at 570 nm with a microplate reader. The viability of BV2 cells was calculated with the following formula: % Viability = (ODexperiment/ODcontrol) × 100%.

### 2.4. Wound Healing Assay

BV2 cells were seeded on 6-well plates and kept in normal growth medium. Cells were allowed to incubate at 37°C in an atmosphere of 5% CO_2_ overnight until confluence. In each well, a single uniform scratch was made along the centre of each monolayer using a sterile pipette tip; the wells were then washed twice with PBS to remove detached cells. LPS and salidroside at indicated concentrations were added to the cells immediately and left for 12 h. The scratch images of the wounded areas were captured at 12 h after incubation with an inverted digital phase contrast microscope. For each well, at least five pictures were taken at a magnification of 100x after scraping. The cell migration ability was quantitatively confirmed by the ratios of wound closure analyzed using Image J software.

### 2.5. Transwell Cell Migration Assay

A transwell (8 *μ*m pore size; Costar, Corning, USA) assay was used to further analyze cell migration according to the manufacturer's instructions. Cells at the density of 5 × 10^4^/mL were placed in the upper chambers in serum-free media, and the lower chambers were added with 0.6 mL 1 *μ*g/mL LPS, 150 *μ*M salidroside, LPS + salidroside, or DMEM as negative control. Cells were allowed to transmigrate into the lower chamber following incubation for 24 h at 37°C; nonmigrating cells on the upper chamber surface were removed. The membranes were fixed with 4% paraformaldehyde in PBS for 30 min and stained with crystal violet in PBS for 15 min. The representative images (magnification, ×100) were randomly taken by an inverted microscope. The cells that migrated to the bottom of the filter were solubilized by cell dissociation solution and transferred to the plate, which was read at 540 nm with microplate reader. Each experimental group was repeated three times.

### 2.6. Measurement of MCP-1, MIP-1*α*, and IL-8

The chemokine expression levels of MCP-1, MIP-1*α*, and IL-8 were measured by enzyme-linked immunosorbent assay (ELISA) kit (R & D Systems, Minneapolis, MN) according to the manufacturer's instructions. In brief, BV2 microglial cells were plated in 24-well plates and stimulated with the indicated concentrations of LPS and/or salidroside for 12 h. The culture-medium supernatants were collected for determination of chemokines concentration at the absorbance at 450 nm using an ELX-808 Microelisa reader (Bio-Tek Inc., Winooski, VT).

### 2.7. RT-PCR (Reverse Transcription-Polymerase Chain Reaction)

The total RNA was isolated using TRIzol (Invitrogen, CA, USA) according to the manufacturer's instructions. RNA (2 *μ*g) was reverse transcribed using the SuperScript First-Strand Synthesis System (Invitrogen, CA, USA). Single stranded cDNA was amplified by PCR with primers for MCP-1 (sense: 5′ ATGCAGGTCCCTGTCATGCTTC 3′, antisense: 5′ TTCTG ATCTCATTTGGTTCC GA 3′, 294 bp); MIP-1*α* (sense: 5′ CCTTGCTGTTCTTCTCTGTACC 3′, antisense: 5′ TCAGTGATGTATTCTTGGACCC 3′, 240 bp); IL-8 (sense: 5′ TACGA TGTCTGTGTATTCAGGAA 3′, antisense: 5′ TTGTGAGCTAAATCAG CAAAGTG 3′, 361 bp); and *β*-actin (sense: 5′ GAGAGGGAAATCGTGCGTGACA 3′, antisense: 5′ ACCCAAGAAGGAAG GCTGGAAA 3′, 192 bp). Thermal cycling conditions were applied: *β*-actin, 20 cycles of denaturation at 94°C for 30 s, annealing at 57°C for 30 s and extended at 72°C for 30 s; for other genes, initial denaturation at 95°C for 2 min, followed by 25 cycles of denaturation at 95°C for 1 min, annealing at 58°C for 1 min and extended at 72°C 30 s. Amplification PCR products were separated by 1% agarose gel electrophoresis and visualized by ethidium bromide staining. *β*-actin was used as an internal control to evaluate relative gene expression.

### 2.8. Protein Extraction and Western Blot

Total cells were harvested after treatment, washed twice with ice-cold PBS, and gently lysed for 15 min in 100 *μ*L radioimmunoprecipitation buffer containing protease inhibitors PMSF. Lysates were centrifuged at 12,000 g at 4°C for 15 min to obtain the supernatants for further analysis. Protein concentration was determined by the bicinchoninic acid method (BCA). Equal amounts of protein samples were denatured in gel-loading buffer at 100°C for 5 min and were separated electrophoretically using 10 or 12% SDS-PAGE and then the gel was transferred to 0.45 *μ*m polyvinylidene fluoride (PVDF: Millipore, Bedford, MA). The membranes were blocked with the use of 5% milk in PBST for 1 h and probed overnight with primary antibodies followed by incubation with horseradish peroxidase-linked secondary antibodies (1 : 1000) at 4°C for additional 1 h. The immunobands were visualized by chemiluminescence- (ECL-) plus (GE, RPN2132) for 1 min, and the intensity was measured using Image J software. *β*-actin was served as an internal control.

### 2.9. Statistical Analysis

Values were presented as mean ± standard error of the mean (SEM). Statistical analyses were conducted with one-way ANOVA using SPSS software (Version 12.0, SPSS Inc., Chicago, IL, USA). Differences were considered statistically significant at *P* < 0.05 for all comparisons.

## 3. Results

### 3.1. Salidroside Did Not Affect Cell Viability in Cultured Microglia

**Figure 1:**
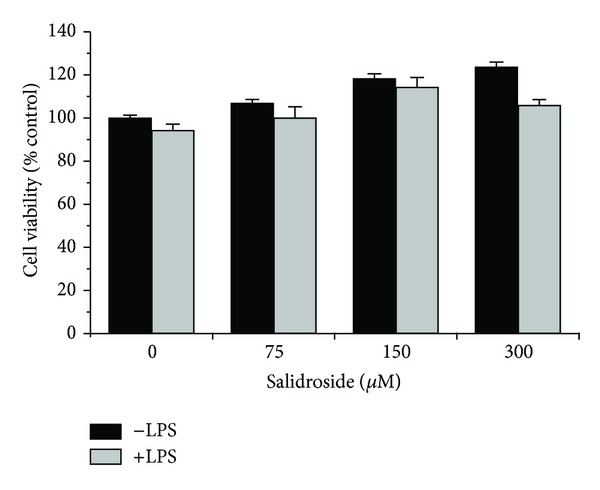
Salidroside does not affect cell viability of BV2 microglial cells. Cytotoxic effect of salidroside on cultured BV2 cell exposure to LPS. BV2 cells were treated with LPS in the presence and absence of salidroside at the concentration of 75, 150, and 300 *μ*M for 24 h. The cell viability was expressed as the percentage of surviving cells compared with control cells by using MTT assay. The data are presented as means ± SD of three independent experiments (*n* = 3).

MTT assay was used to determine the cytotoxic potential of salidroside on the viability of BV2 microglial cells. After treatment with LPS (1 *μ*g/mL) in the presence or absence of salidroside at various concentrations (75–300 *μ*M) for 24 h, salidroside at different concentrations alone caused no apparent cytotoxicity on cultured BV2 cells. Furthermore, salidroside in the presence of LPS also indicated no cytotoxic effects on BV2 cells (Figure 1). Therefore, salidroside levels at the concentration of 75, 150, and 300 *μ*M were chosen for protecting BV2 cells in all subsequent experiments unless otherwise stated.

### 3.2. Salidroside Inhibited the Migratory Capacity of BV2 Exposed to LPS

**Figure 2:**
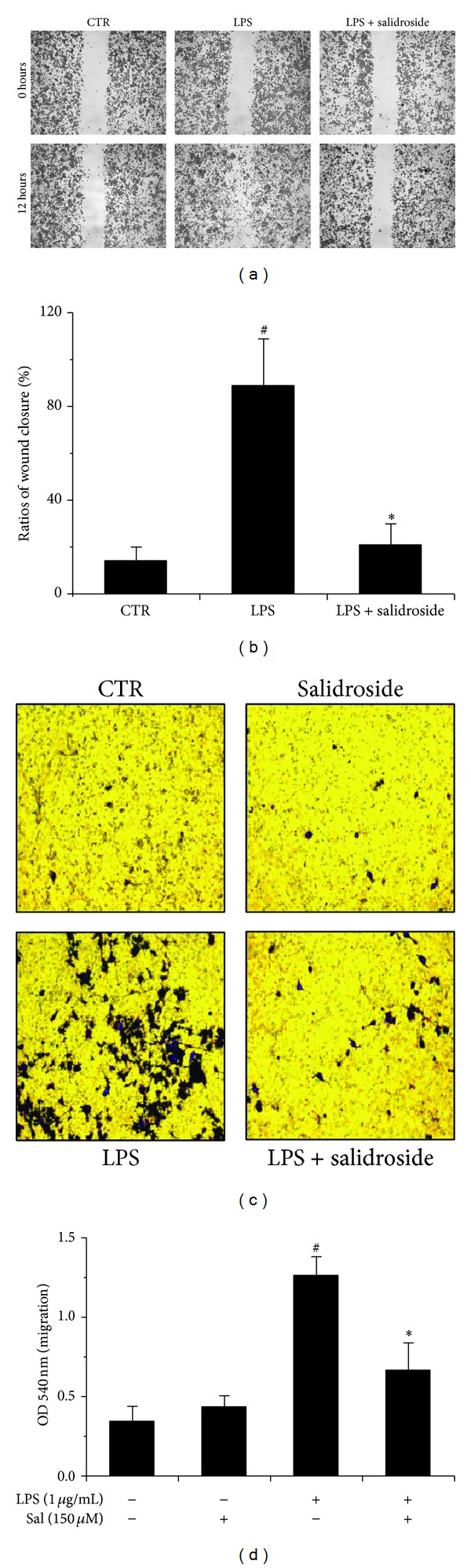
Salidroside exhibits antimigratory property in BV2 microglial cells. ((a), (b)) The wound scratch assay was carried out to analyze motility of LPS-activated BV2 cells. Representative phase contrast images show the width of scratch captured at 12 h after scraping. The cell migration distances were determined with the ratios of wound closure. ((c), (d)) Determination of BV2 cell migration by transwell migration assay. Cells were fixed, stained, and examined microscopically in five random fields at 100x magnification after 24 h treatment and then were harvested and read with microplate reader. Absorbance correlating with the number of cells invaded expressed as mean ± SD (*n* = 3). ^#^ *P* < 0.05 versus the control group; *P < 0.05 versus LPS group.

Scratch wounds were inflicted on cells treated by LPS alone or with salidroside for 12 h. In this study, we set out to deserve closure of the wound which depends on cell migration from the scratched edge to determine whether salidroside affects the migration potential in LPS-induced BV2 cell line. The difference of surface area generated by the wound between the groups was observed after 12 h of treatment (Figure 2(a)). We observed that there were few cell migrations in the control group, which made no influence on microglial migration, while LPS group exhibited highly BV2 cell migratory potential, which was significantly reduced by salidroside (150 *μ*M) (Figure 2(b)).

Accordingly, as shown in Figures 2(c) and 2(d), the transwell migration assay showed that a greater number of viable cells migrated into the lower chamber of the transwell following treatment with LPS compared with that of the control group. However, the LPS + salidroside group demonstrated a fewer migrated cells than that of LPS group. These data suggested that migratory ability of BV2 cells was significantly suppressed by salidroside confirmed further by quantitative analysis.

### 3.3. Salidroside Attenuates the LPS-Induced Chemokines Secretion and Expression in Microglia

**Figure 3:**
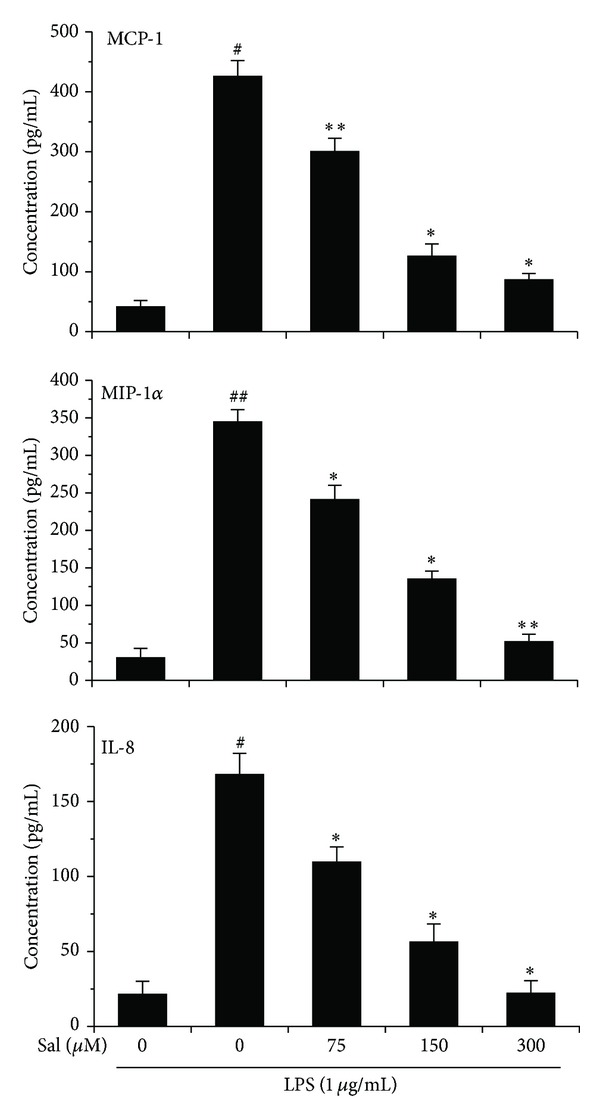
Salidroside suppresses LPS-induced chemokine production in BV2 microglial cells. Cells were incubated with salidroside at various concentrations and/or LPS for 12 h. The amounts of MCP-1, MIP-1*α*, and IL-8 production in the supernatant were measured using ELISA. The OD values are the mean ± SD of three separate experiments. ^#^ *P* < 0.05, ^##^ *P* < 0.01 versus the control group; *P < 0.05, **P < 0.01 versus LPS group.

ELISA and RT-PCR assay was carried out to examine the effects of salidroside on protein and mRNA expressions of chemokines in LPS-induced BV2 cells after 12 h. We selected three chemokines that are associated with cell migration such as MCP-1, MIP-1*α*, and IL-8. As seen in Figure 3, BV2 cells subjected to LPS for 12 h at 1 *μ*g/mL presented significant higher chemokine secretion than those in the control group; however, all concentrations of salidroside at 75, 150, and 300 *μ*M treatments reduced the protein expression significantly in a dose-dependent manner, as assessed by ELISA technique.

**Figure 4:**
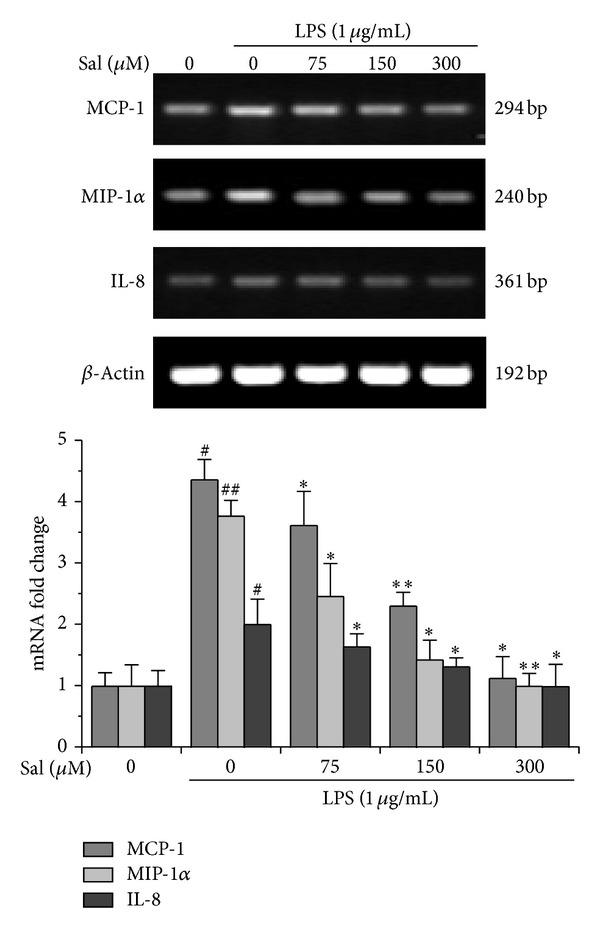
Salidroside inhibits LPS-induced migratory transcripts in BV2 cells. BV2 Cells were coincubated with LPS (1 *μ*g/mL) and salidroside at indicated concentrations mentioned previously. The total RNA was isolated, and MCP-1, MIP-1*α*, and IL-8 were performed by RT-PCR analyses. Expression was normalized against the internal control *β*-actin. The representative RT-PCR images were shown and the data were expressed as the mean ± SD of three independent experiments (*n* = 3). ^#^ *P* < 0.05, ^##^ *P* < 0.01 versus the control group; *P < 0.05, **P < 0.01 versus LPS group.

Similarly, LPS upregulated the mRNA levels of these three chemokines compared to those of the normal control group; however, salidroside significantly suppressed the expression of chemokine gene transcripts in LPS-stimulated BV2 cells, displaying a dose-dependent pattern (Figure 4).

### 3.4. NF-*κ*B and MAPK Signaling Pathway Was Involved in Antichemotaxis of Salidroside Against LPS-Induced Microglial Cell Activation

**Figure 5:**
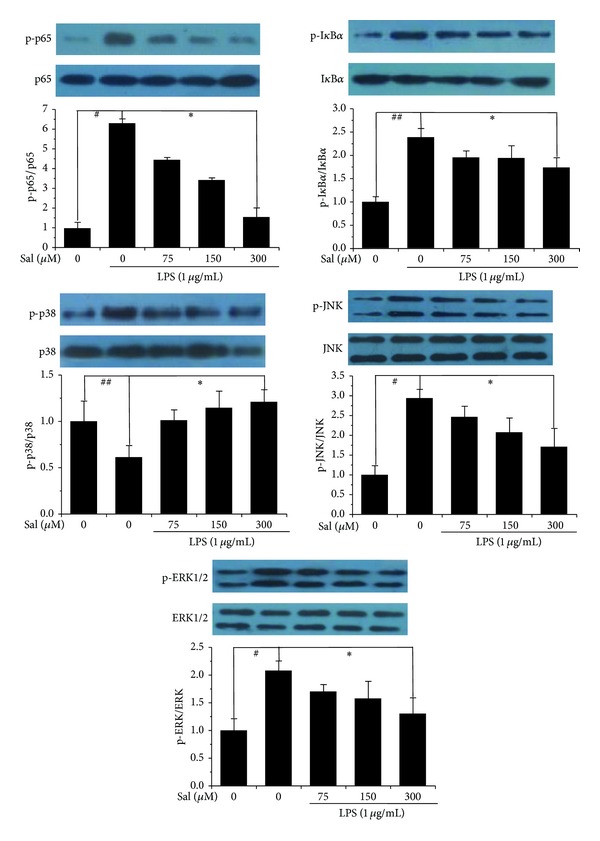
The NF-*κ*B and MAPK pathway were involved in the cell motility suppression of salidroside in LPS-induced BV2 cells. Cells were treated with LPS and salidroside at indicated concentrations and time. The protein level relating to NF-*κ*B and MAPK pathway was measured by Western-blotting analysis. The representative image was from one of three repeated experiments. Expression of all proteins was quantified by densitometry analysis. Data were shown as the mean ± SD. ^#^ *P* < 0.05, ^##^ *P* < 0.01 versus the control group; *P < 0.05 versus LPS group.

In order to investigate the mechanism underlying the inhibitory effect of salidroside, we used LPS to activate NF-*κ*B and MAPK pathway measured by Western-blotting assay. The results showed significant increases in the degradation of I*κ*B*α* and phosphorylation of p65, I*κ*B*α*, P38, JNK, and ERK1/2 in the LPS-treated group as compared to the control group, whereas treatment with salidroside at indicated concentrations exhibited significant reduction of I*κ*B*α* degradation and all the phosphorylated protein expression in a dose-dependent pattern (Figure 5). The measurement revealed that salidroside attenuates the LPS-induced BV2 cell activation, perhaps partly, by blocking the NF-*κ*B and MAPK signaling simultaneously.

## 4. Discussion

Cerebral ischemia or stroke remains a leading cause of death and long-term disability in the aged population and there are no efficient curative treatments. Cerebral ischemia leads to brain injury via a complex series of pathophysiological events, and inflammation has been implicated as a key contributor to the pathophysiology of ischemic stroke [4]. The main mechanism contributing to inflammation in ischemic stroke is activation of microglia, which can further propagate neuronal death. Microglia are considered a specialized form of macrophage residing in the CNS [5]. Microglia excess activation is essential to the disease progression resulting in an induction of neurotoxic proinflammatory mediators (cytokines and chemokines) that can amplify neuronal damage. Therefore, it provides an attractive research concern to develop new multitargeting drugs or agents and investigate the molecular mechanism of neuroprotection.

Salidroside is a compound of plant origin with a definite chemical structure of phenol glycoside extracted from the root of* Rhodiola rosea*. Previously, it has been shown that it possesses a broad spectrum of pharmacological properties, including anti-inflammation [3, 6], antioxidation [7, 8], and protective effects against neuronal death [9–13]. Moreover, salidroside has been previously shown to have neuroprotective and antineuroinflammatory effect in neurodegenerative diseases (such as AD and stroke) [3, 14], demonstrating that salidroside may have therapeutic potential for neuroinflammation. It had been reported that salidroside exhibited the inhibitory effect on the inflammatory cytokine production in LPS-charged BV2 cell [3], in order to have a new insight into the potential neuroprotective functions peculiar to salidroside against cerebral ischemia; we further used the LPS-induced BV2 cell model to investigate the antichemotaxic effects of salidroside and the possible involvement of the NF-*κ*B and MAPK signaling pathway.

In the present study, MTT assay confirmed that salidroside treatment did not affect the cell viability at a certain concentration (75–300 *μ*M) with or without LPS, suggesting that inhibition of LPS-induced BV2 cell motility was not resulting from a cytotoxic action of salidroside. Subsequently, the cell migration was evaluated by stretch-induced injury and transwell migration assay* in vitro*. As shown in Figures 2(a)–2(d), wound closure was significantly inhibited when cells were incubated with salidroside at concentrations of 150 *μ*M. In addition, the treatment with 150 *μ*M salidroside also alleviated the ability of the BV2 cells to migrate to the lower side of the well following LPS stimulation. Both results showed that salidroside treatment greatly inhibited the LPS-induced cell motility in BV2 cells.

Moreover, ELISA and RT-PCR analysis showed that LPS stimulation led to an increased level of migration-related genes and proteins in BV2 cells as compared to control, while salidroside treatment suppressed the LPS-induced elevation in chemokine expression and the suppression exhibited a dose-dependent pattern (Figures 3 and 4). The results further suggested that neuroprotection of salidroside against LPS-treated cell migration was, at least partly, attributable to the regulation of chemokine production.

To further explore the possible mechanisms underlying the inhibitory effects of salidroside on LPS-stimulated migrated BV2 cells, we further investigated the effect of salidroside on the involving signaling transduction pathways, such as NF-*κ*B and MAPK signaling. The transcription factor NF-*κ*B, widely expressed in the nervous system, plays a leading role in LPS-induced inflammatory responses [15]. In this study, we noted that NF-*κ*B activation was dramatically induced by LPS stimulation, while salidroside regulates microglial activation by inhibiting the I*κ*B*α* degradation and phosphorylation of I*κ*B*α* and p65, a subunit of NF-*κ*B. In addition, MAPK pathway has been proposed to take part in the cellular migration of postischemic inflammation [16]. Consistent with a previous report [17], our results suggest that LPS significantly induces the phosphorylation of ERK1/2, JNK, and p38; however, treatment with salidroside downregulated the LPS-induced phosphorylation of these three components of MAPK family, suggesting that a salidroside-mediated MAPK pathway is another effecter in LPS-induced chemokine gene regulation. Thus, our observations implicated that salidroside exerted its neuroprotective effects on LPS-induced cell chemotaxis via MAPK and NF-*κ*B pathway.

To conclude, our data first indicates that salidroside treatment* in vitro* ameliorates cell migration in cultured BV2 cells, and its antichemotaxic effect might be associated with the modulation of migration-related protein and their regulatory genes mediated by NF-*κ*B activation and MAPK pathway. Together these results indicate that salidroside is a potent neuroprotectant candidate for treating neurodegenerative diseases (such as stroke) associated with microglial activation.

## Abbreviations

CNS: Central nervous system
LPS: Lipopolysaccharide
MTT: 3-(4,5-Dimethylthiazol-2-yl)-2,5-diphenyl-tetrazolium bromide
DMEM: Dulbecco's modified Eagle's medium
FBS: Fetal bovine serum
PBS: Phosphate buffer saline
MCP-1: Monocyte chemoattractant protein-1
MIP-1*α*: Macrophage inflammatory protein-1 alpha
IL-8: Interleukin-8
MAPKs: Mitogen-activated protein kinases
JNK: c-Jun N-terminal protein kinase;
ERK1/2: Extracellular signal-regulated kinase 1/2
NF-*κ*B: Nuclear factor kappa B
I*κ*B*α*: Inhibitor *κ*B-*α*.
